# Oxidative stress in lens in vivo: Inhibitory effect of caffeine. A preliminary report

**Published:** 2010-03-23

**Authors:** SD Varma, KR Hegde, S Kovtun

**Affiliations:** 1 Department of Ophthalmology & Visual Sciences, University of Maryland School of Medicine, Baltimore, MD; 2 Department of Biochemistry and Molecular Biology, University of Maryland School of Medicine, Baltimore, MD

## Abstract

**Purpose:**

Experiments have been conducted to study the hypothesis that caffeine would inhibit reactive oxygen species induced oxidative stress in the lens in vivo, with implications of attenuating or preventing cataract formation.

**Methods:**

Oxidative stress was directly induced by administering 24% galactose diet to young adult rats. The treated group was fed a diet containing 24% galactose + 1% caffeine. Oxidative stress inflicted to the lens was assessed by measurement of glutathione (GSH) depletion and observing the status of lens clarity.

**Results:**

Caffeine administration was found to minimize the loss of GSH. This was also associated with a better maintenance of lens transparency as compared to the untreated galactosemic group.

**Conclusions:**

The studies demonstrate that caffeine could be helpful in inhibiting oxidative stress in the lens with the consequence of attenuating cataract formation.

## Introduction

Caffeine (1,3,7-trimethylxanthine) is one of the common ingredients of many beverages such as coffee, tea and various colas. It is also widely used medically, as a CNS, respiratory and cardiac stimulant, smooth muscle relaxant, analgesic and as a diuretic [1]. The stimulatory effect of caffeine in the nervous system has been attributed to its competitive binding with certain pre-synaptic adenosine receptors and consequently abolishing the inhibitory but regulatory effect of adenosine on release of various neurotransmitters. Adenosine binding to its receptor in the pre-synaptic terminal has the effect of limiting calcium ion penetration through the cell membrane, and thus inhibiting neurotransmitter release at the synapses [2]. The basic effect of caffeine binding to the adenosine receptors is therefore to overcome the inhibition of calcium passage through the cell membranes and consequently facilitate the release of stimulatory neurotransmitters such as epinephrine. Its action on stimulation of respiratory, cardiac and skeletal muscles has also been suggested to be exerted by an increase in the cytosolic calcium by causing its de-sequestration from the sarcoplasmic reticulum [3-5]. It does so also by facilitating a direct calcium penetration through the cell membrane [3]. In addition to its neural and muscular effects, caffeine has been shown to have several other physiologic effects such as a general metabolic stimulation [6-8] and thermogenesis [9]. The latter is considered useful in improving sports performance. Most of the latter effects are suggested to be associated with an increase in the cellular level of c-AMP induced by virtue of its ability to inhibit adenosine 3′, 5′- monophosphate phosphodiesterase activity [10].

In addition to the above actions, more recent observations suggest that it can also act as an antioxidant. The suggestions are largely based on chemical studies showing it to be able to scavenge reactive oxygen species (ROS), particularly the hydroxyl radical (OH^·^), known to be generated in the body by irradiation with various electromagnetic frequencies such as exposure to UV, as well as by many ambient physiologic reactions involving oxygen utilization [11-13]. The generation of these radicals is also enhanced in the tissues in many pathological conditions induced by aging and certain diseases. The interaction of OH^·^ with caffeine results in its oxidative de-methylation, generating partially N-methylated xanthines such as theobromine, paraxanthine, and theophylline [14,15]. In addition, OH^·^ also leads to the fission reactions at the double bonds producing methylated parabanic acid [16]. However under milder oxidative conditions, as prevalent physiologically, the prominent reaction is the generation of 8-hydroxy caffeine (1,3,7-trimethyl-8-hydroxyxanthine), which is structurally analogous to uric acid derived from xanthine [12,17]. The physiologic usefulness of the above reactions is strongly indicated by the radioprotective effects of caffeine against radiations such as the UV irradiation. Additionally, it has been shown to prevent Fenton’s reaction-induced oxidation of glutathione [11], a major antioxidant reserve in many tissues, including those of the eye. However, studies on examination of the possible protective effect of caffeine against ROS induced oxidative stress at the level of cell and tissue culture as well as in vivo are yet very limited, specially in the eye where oxidative stress has been implicated in the genesis of diseases such as cataracts [18-22] and retinal degenerations [23-25]. Recently we have shown that oxidative damage to the lens in organ culture inflicted by UV exposure or by trace metals such as iron, can be significantly prevented by caffeine [26,27]. Preliminary data presented here strongly suggest the possibility that caffeine would be effective in preventing oxidative stress to the lens in vivo also. Such stress was induced by feeding high galactose diet to young adult rats. The antioxidant effect of caffeine was assessed by measuring its ability to prevent loss of tissue glutathione and preserve lens transparency.

## Methods

All chemicals and reagents were purchased from Sigma Chemical Co. (St. Louis, MO) Rats were purchased from Harlan Laboratories and used in accordance with ARVO guidelines and as approved by the institutional animal care and use committee (IACUC).

Young Sprague Dawley rats weighing about 45 g were used. The control group was fed a powdered laboratory chow containing 24% galactose ad lib. The experimental group received the same diet but also containing 1% caffeine. Since the cataract process was found to set in within a couple of days apparent ophthalmoscopically, they were euthanized on day 4, lenses isolated, weighed, photographed, and used for determination of glutathione (GSH). Dulcitol was measured by method of West and Rapoport [28]. The level of blood galactose in both the groups was 16±2 mM, determined using a Boehringer-Manheim kit provided by Roche (Cat # 10 176 303 035; Manheim, Germany). The measurement is based on spectrophotometric determination of nicotinamide adenine dinucleotide, reduced (NADH) produced from nicotinamide adenine dinucleotide (NAD) by D-Galactose dehydrogenase dependent oxidation of galactose. Blood caffeine varied between 0.06 to 0.012 mM measured in the hospital clinical laboratory.

### Measurement of GSH and ATP

The isolated lenses were homogenized in 0.5 ml dH_2_O and centrifuged. An aliquot of the supernatant was used for the determination of ATP as described previously [26]. Subsequently trichloroacetic acid was added to a final concentration of 5% and supernatant obtained by centrifugation of the sample. This supernatant (100 µl) was then mixed with 300 µl of 0.6 M Na_2_HPO_4_. This was followed by the addition of 100 µl DTNB (5, 5′dithio-bis-2-nitrobenzoic acid) reagent [29]. The resulting yellow color was read spectrophotometrically at 412 nm. DTNB reagent was prepared by dissolving 4 mg DTNB and 100 mg trisodium citrate in 10 ml dH_2_O. GSH standards were also run simultaneously.

## Results

**Figure 1:**
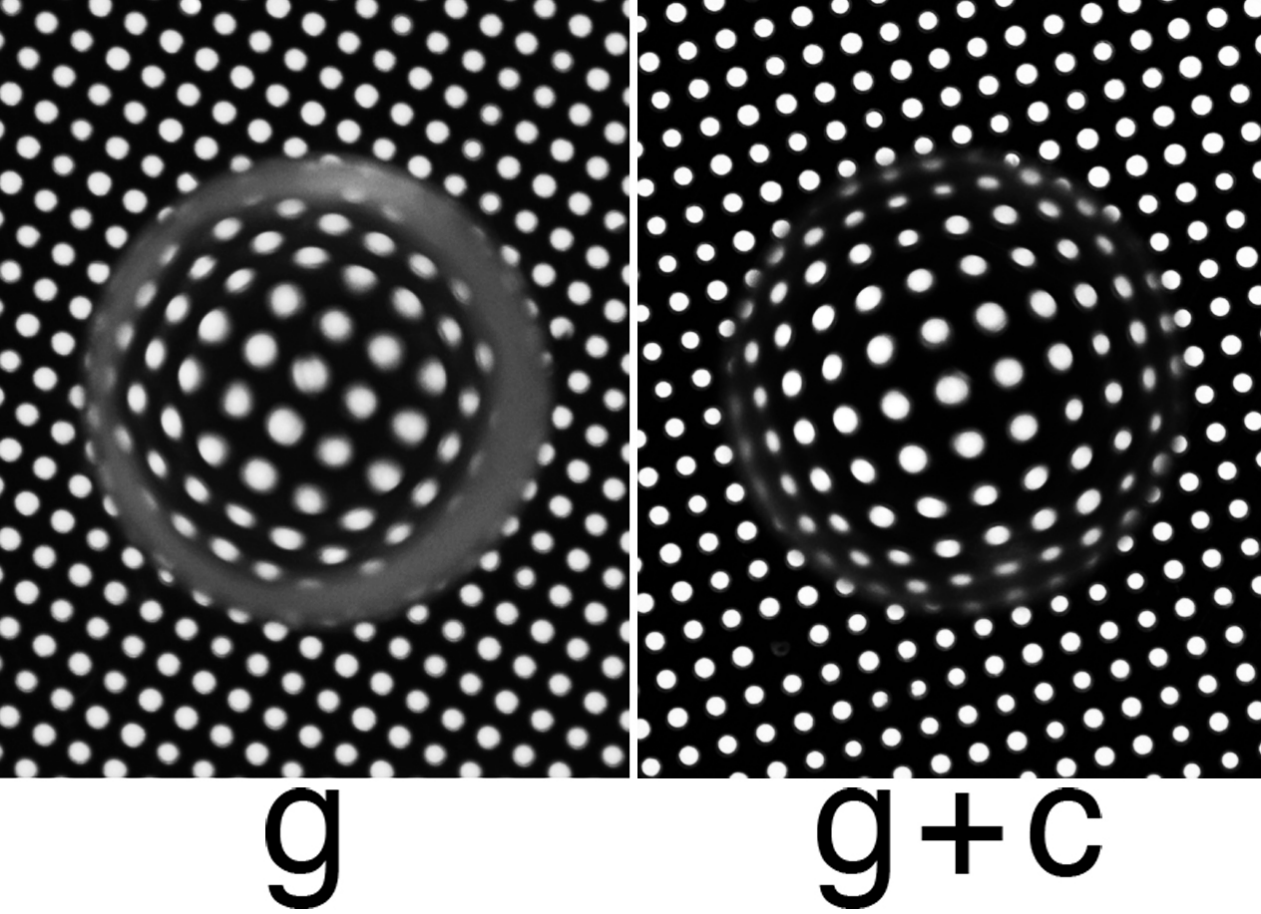
Inhibition of cataract formation by caffeine. Trans-illumination photographs of representative lenses isolated from rats fed 24% Galactose diet without and with 1% Caffeine: Peripheral ring opacity (cortical cataract) was highly apparent in the untreated group (g), representing the early stage of galactose cataract. The lenses in the treated group receiving caffeine were transparent (g+c).

The results are described in Figure 1 and in Table 1, Table 2, and Table 3, the data in each table representing 8, 6, and 8 animals, respectively. Since the galactose induced opacities in the caffeine untreated animals were similar in both the eyes, they were treated as one observation, treating the contralateral lenses as duplicates for biochemical analysis. Lenses in the caffeine treated group had no opacity. As summarized in Table 1, caffeine inhibited the decrease in the content of lens GSH in galactose fed animals. The level of this tripeptide in the control normal rats was about 6 µmoles/g as also reported previously [30]. The level in the galactose fed animals was near 0.9 µmoles/g. The drop in GSH level was hence very substantial as reported previously also [31]. The level in the galactose plus caffeine group was about 5 times higher. Hence the decrease in GSH in the lenses of caffeine fed animals was much less than that observed in the lenses of rats fed the galactose diet without caffeine. ATP levels were also maintained in the caffeine treated group. Previous electron paramagnetic resonance (EPR) studies have demonstrated that this loss is attributable to the generation of OH^·^ during GSH oxidation caused by the excessive diversion of NADPH toward dulcitol synthesis, instead of its use in reducing GSSG to GSH [32]. As shown in Table 2, there was no decrease in dulcitol level caused by caffeine feeding, proving again that the loss of GSH, at least in the early stages of cataracts is primarily due to its ROS dependent oxidation. The physiologic significance of caffeine feeding was further apparent by the status of lens clarity and opacification (Figure 1). Visually, the lenses in the caffeine free group were hazy in general appearance. In addition they had highly apparent cortical cataracts. This was not the case with the lenses in the caffeine group where the lenses were transparent with no visible opacities. This was assessed also by Trans- illumination photography. As shown in Figure 1, lenses in the caffeine group were significantly more transparent with no cataractous changes. On the contrary, highly obvious cortical cataract was noticeable in the untreated galactosemic group. As previously reported, formation of cataract in galactosemic animals is associated with a significant increase in tissue weight because of osmotic hydration due to dulcitol accumulation. However, the dulcitol levels were not affected by caffeine-albeit it was noticeable higher in certain lenses of the caffeine group. Despite this, as shown in Table 3, caffeine feeding was found to inhibit the expected increase in lens weight, implying that the inhibition of hydration observed with caffeine treatment could be due to the prevention of oxidative damage inflicted by oxidative loss of GSH and damage to membrane Na^+^-K^+^ ATPase. The latter has been previously demonstrated by histochemical studies [33] as well as in lens culture studies [34,35].

**Table 1:** Effect of caffeine on levels of GSH and ATP in the lenses.

**Groups**	**GSH (µmoles/g wet wt. of lens)**	**ATP (µmoles/g wet wt. of lens)**
Control	6.15±0.15	1.3±0.05
Galactose	0.9±0.07*	1.0±0.055
Galactose + caffeine	5.01±0.05 **	1.35±0.005

Rats weighing 40–45 g were fed a 24% galactose diet with or without 1% caffeine. GSH was then determined in the TCA extracts of the tissue after its neutralization with Na_2_HPO_4_ and reacting it with DTNB reagent as described in the text. The values marked with an asterisk are significantly lower than the corresponding controls. The values marked with a double asterisk are significantly higher than those marked with an asterisk and are significantly closer to the controls, p<0.05, n=8 (number of animals).

**Table 2:** Effect of caffeine on lens dulcitol.

**Group**	**Dulcitol (µmoles/g wet wt.)**
Galactose	17.2±2.5
Galactose + Caffeine	19.6±6

Rats weighing 40–45 g were fed a 24% galactose diet without or with 1% caffeine. There were then euthanized, lenses dissected out and as described, transferred on a Millipore grid and photographed by trans-illumination using 50 W daylight tube. Subsequently they were used to prepare a protein free filtrate by homogenization with Nelson-Semoygi reagent and centrifugation. Dulcitol was determined as described in the text. Values are expressed as mean±s.d., n=6 (number of animals).

**Table 3:** Effect of caffeine against galactose induced lens hydration as indexed by weight gain.

**Groups**	**Lens Weight (mg)**	**Clarity**
Control	18.5±0.01	Clear
Galactose	21.0±0.35*	Hazy
Galactose + caffeine	16.95±0.01**	Clear

Values marked by an asterisk were noticeably higher than the controls. Values marked with a double asterisk were closer to the controls, p<0.05, n=8 (number of animals).

## Discussion

Several previous studies have suggested that intraocular generation of oxygen free radicals such as superoxide and hydroxyl radicals and consequent oxidative stress is one of the significant factors involved in the genesis of cataracts associated with aging, UV exposure, and many diseases such as diabetes. Treatment with certain scavengers of reactive oxygen is expected to thwart the oxidative stress component of cataract formation. It is hypothesized that this can be achieved by use of ROS scavengers derived nutritionally, such as ascorbate [36,37], tocopherols [38,39], and bioflavonoids [40]. However, they get oxidized during food processing as well as during cooking, unlike caffeine which is present in substantial amounts even after heating associated with initial processing of tea leaves and coffee beans as well as in hot water used while preparing the drink proper. Hence its property of scavenging ROS and consequent antioxidant activity still remains maintained. Although substantial number of studies already exist on the medicinal and physiologic effects of caffeine in other systems, such as its effect in neural and muscular systems and the tissue metabolism and bioenergetics, studies on its physiologic effects in the eyes are as yet largely lacking, except our recent studies showing that it can potentially prevent oxidative damage to the lens with implications against cataract development. This has been suggested on the basis of in vitro studies showing it ability to inhibit oxidative damage to lens inflicted by UV irradiation as well as by the radicals produced by enriching the culture medium with iron [26,27]. The primary aim of this investigation was hence to study if it could be useful in preventing oxidative stress to the lens in vivo also. This was done in the galactosemic rat model where the link between oxyradical generation with cataract formation has now been more convincingly apparent [32,38]. Treatment with caffeine was found to inhibit the loss of glutathione as well as ATP. The decrease in the level of these components in the caffeine untreated galactose fed group is similar to that reported earlier [31,41]. The significance of the effectiveness of caffeine against ROS damage was apparent physiologically also. Apart from the maintenance of lens clarity, other features of cataract formation such as lens hydration, was also prevented significantly by treatment with caffeine. Interestingly this effect was not found to be associated with any inhibition of lens dulcitol production. In an ongoing study we have seen that the lenses of galactosemic animals given caffeine retain their clarity even till three weeks. Further experiments with different levels of caffeine are hence considered desirable, including such studies with the diabetic model and administering caffeine also topically. The present preliminary results showing an in vivo effectiveness of caffeine indeed lay the foundation for further mechanistic studies involving other modes of caffeine effect, such as its relationship with possible maintenance of a higher level of cyclic nucleotides, attributable to its inhibitory effect on phosphodiesterase.
